# Preparation and Certification of a New Salvianolic Acid A Reference Material for Food and Drug Research

**DOI:** 10.1007/s13659-020-00236-2

**Published:** 2020-04-18

**Authors:** Dezhi Yang, Bin Su, Yancai Bi, Li Zhang, Baoxi Zhang, Junke Song, Yang Lu, Guanhua Du

**Affiliations:** 1 Beijing City Key Laboratory of Polymorphic Drugs, Center of Pharmaceutical Polymorphs, Institute of Materia Medica, Chinese Academy of Medical Sciences and Peking Union Medical College, Beijing, 100050 People’s Republic of China; 2 Soteria Pharmaceutical Co., Ltd., Laiwu, 271100 People’s Republic of China; 3 Beijing City Key Laboratory of Drug Target and Screening Research, National Center for Pharmaceutical Screening, Institute of Materia Medica, Chinese Academy of Medical Sciences and Peking Union Medical College, Beijing, 100050 People’s Republic of China

**Keywords:** Certified reference material, Salvianolic acid A, Mass balance method, qNMR, High resolution mass spectrum

## Abstract

**Abstract:**

Salvianolic acid A (Sal A), a water-soluble ingredient in Danshen, has various biological activities. Sal A and its impurities have similar physical and chemical properties, as well as strong reducibility; therefore, they are difficult to prepare and purify. In this study, high-purity Sal A was obtained by purification of sephadex chromatography and preparative chromatography. Furthermore, HPLC–DAD tandem ECD and HPLC–DAD tandem MS methods were used for non-volatile organic impurity analysis, ICP-MS method was used for non-volatile inorganic impurities and mass balance method and quantitative nuclear magnetic resonance were employed to certify the product. The structures of Sal A and its relative impurities were validated by nuclear magnetic resonance spectroscopy and mass spectrometry, and their contents were quantified as well. Following the principles of ISO Guides 34:2009 and 35:2005, a Sal A reference material was certified, covering homogeneity studies, stability studies, characterization, and uncertainty estimations.

**Graphic Abstract:**

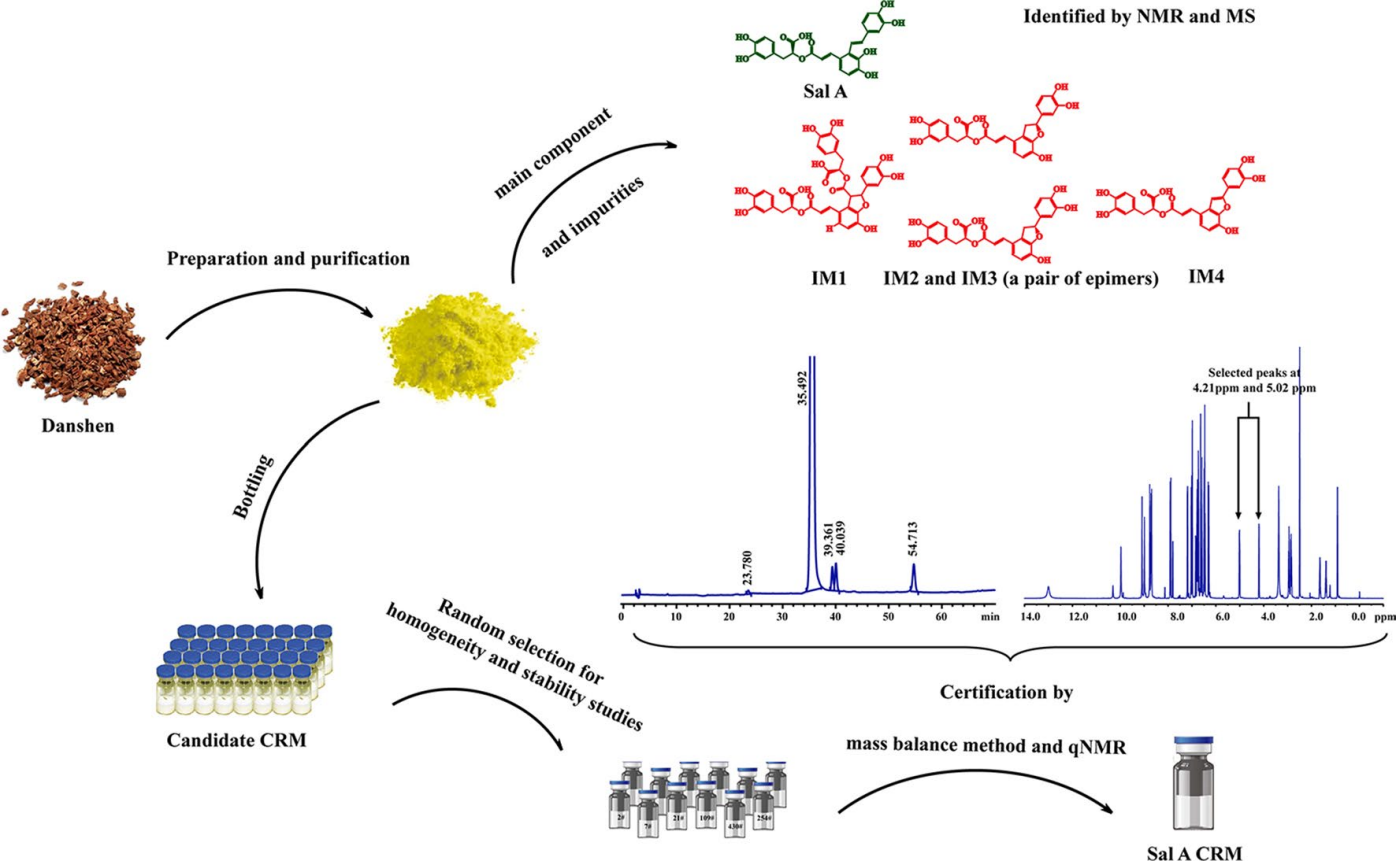

**Electronic supplementary material:**

The online version of this article (10.1007/s13659-020-00236-2) contains supplementary material, which is available to authorized users.

## Introduction

Salvianolic acid A ((2*R*)-3-(3,4-dihydroxyphenyl)-2-[(*E*)-3-[2-[(*E*)-2-(3,4-dihydroxyp-henyl) ethenyl]-3,4-dihydroxyphenyl] prop-2-enoyl] oxypropanoic acid, Sal A) is one of the major water-soluble phenolic acids extracted from *Salvia miltiorrhiza* Bunge (Danshen) [1], which is a highly versatile oriental herbal drug. As a major effective constituent of Danshen, Sal A has various biological and pharmaceutical activities due to its strong antioxidant activity, moreover, the treating potential of Sal A on diabetic peripheral neuropathy has gathered interest [2]. A new drug derived from Sal A is currently in phase I clinical trials. Other bioactivities of Sal A include anti-thrombosis [3], anti-fibrosis (Liu et al. [4]), anti-cancer [5], and myocardial protection [6]. Therefore, Sal A has extensive prospects as a therapeutic agent in drug or a function element in health food.

However, its low content in raw material (approx. 0.01–0.03%) and its strong reducibility make Sal A difficult to be prepared; moreover, other water-soluble phenolic acids, such as salvianolic acid B (Sal B), isosalvianolic acid (Isosal C), salvianolic acid C (Sal C), and isomers of Sal A, have similar physical and chemical properties with Sal A, making them difficult to be purified as well. Therefore, no certified reference material for drug control of Sal A exists in the market. This situation limits the drug developments involving Sal A. In this study, high-purity Sal A was obtained by using sephadex chromatography and preparative high performance liquid chromatography. Moreover, further research on certified reference material (CRM) of Sal A was carried out.

A CRM is a material or substance that has one or more property values being sufficiently homogeneous, stable, and well established enough to be used for calibrating apparatuses, assessing measurement methods, or assigning values to materials (ISO [7, 8]). CRMs are essential tools for guaranteeing the metrological traceability of measurement results to the International System of Units, which assures the accuracy and comparability of results over time and space [9]. In this paper, high performance liquid chromatography-mass spectrometry (HPLC–MS) was used to identify the impurities in the Sal A CRM, and diode array detector (DAD) tandem electrochemical detector (ECD) was used in HPLC analysis. By using preparative chromatography, we obtained five of the identified impurities, and their structures were validated by using nuclear magnetic resonance (NMR) spectroscopy; moreover, the correction factors of these impurities were obtained. Finally, according to the ISO guides 30 (ISO [10]), 34, and 35, the aspects of homogeneity, stability, characterization, and uncertainty estimation were studied. In the characterization, we used the mass balance method, which is regarded as a potential primary method and is recommended by the World Health Organization and European Pharmacopoeia, and International Pharmacopoeia for the establishment of chemical reference standards; the mass balance method is also recommended by the Bureau International des Poids et Mesures for the comparison of national metrology institutes [11]. Meanwhile, quantitative nuclear magnetic resonance (qNMR) spectroscopy has also been employed for Sal A CRM purity measurements, which has emerged as an important tool for certified reference materials to assign purity values in organic molecules [12–14]. Furthermore, the uncertainty evaluations was performed carefully based on the Guide to Uncertainty Measurement (ISO [15]). The certified value of Sal A CRM was 94.7% with an expanded uncertainty of 0.4%. Therefore, the Sal A CRM can be used to validate analytical and quality control methods for new drug and health food developments (Tables 1, 2).

**Table 1:** Equations used in the homogeneity and stability studies of Sal A CRM

Equation	No	Description
$u_{{\text{h}}} = \sqrt {{{\left( {MS_{{{\text{within}}}} - MS_{{{\text{between}}}} } \right)} \mathord{\left/ {\vphantom {{\left( {MS_{{{\text{within}}}} - MS_{{{\text{between}}}} } \right)} n}} \right. \kern-\nulldelimiterspace} n}}$	(1)	*u*_h_ is the uncertainty of homogeneity; *MS*_within_ is the mean square within groups; *MS*_between_ is the mean square between groups; *n* is the number of replicates
$S^{2} = \frac{{\sum\nolimits_{i = 1}^{n} {\left( {Y_{i} - b - aX_{i} } \right)^{2} } }}{n - 2}$	(2)	*S* is the standard deviation of the straight line; *Y*_*i*_ is the purity of the CRM; *X*_*i*_is the time; *a* is the slope; *b* is the intercept; *n* is times of the measurements
$S_{{\text{(b)}}} = \frac{S}{{\sqrt {\sum\nolimits_{i = 1}^{n} {(X_{i} - \overline{X} )^{2} } } }}$	(3)	*S*_(b)_ is the slope uncertainty; *X* is the time
$t_{4}^{0.05} \times S_{{({\text{b}})}}$ compare with $\left| a \right|$	(4)	$t_{4}^{0.05}$ is the critical value of *t*-test where *n* (degree of freedom) is 4 and confidence interval is 0.95
$u_{{{\text{sts}}}} \left( {{\text{temp}}} \right) = u_{{{\text{sts}}}} ({\text{photo}}) = S_{{\left( {\text{b}} \right)}} t$	(5)	*u*_sts_(temp) is the uncertainty of stability at the condition of high temperature; *u*_sts_(photo) is the uncertainty of stability at the condition of strong illumination; *t* is the time of short-term stability study
$u_{{{\text{sts}}}} = \sqrt {u_{{{\text{sts}}}}^{{2}} \left( {{\text{temp}}} \right) + u_{{{\text{sts}}}}^{{2}} ({\text{photo}})}$	(6)	*u*_sts_ is the uncertainty of short-term stability
$u_{{{\text{lts}}}} = S_{{\left( {\text{b}} \right)}} t$	(7)	*u*_lts_ is the uncertainty of long-term stability; *t* is the time of long-term stability study

**Table 2:** Equations used in mass balance method

Equation	No	Description
$P_{{{\text{MB}}}} \% = \left( {1 - x_{{{\text{oi}}}} } \right)\left( {1 - x_{{{\text{mo}}}} - x_{{{\text{re}}}} - x_{{{\text{sa}}}} } \right) \times 100\%$ where $x_{{{\text{oi}}}} = \frac{{\sum\nolimits_{{{\text{i }} = {\text{ }}1}}^{{\text{n}}} {A_{{{\text{oii}}}} } }}{{A_{{{\text{tot}}}} }}$	(8)	*P*_MB_ is the purity of sample determined by MB; *x*_oi_ is the amount of organic impurities; *x*_mo_ is the amount of water; *x*_re_ is the amount of residual solvent; *x*_sa_ is the amount of sulfated ash; *A*_oi_ is the corrected chromatographic peak area of organic impurities. *A*_tot_ is the corrected chromatographic peak area of all compounds
$P_{{{\text{qNMR}}}} \% = x_{{{\text{qNMR}}}} - x_{{{\text{mo}}}} - x_{{{\text{re}}}} - x_{{{\text{sa}}}} \times 100\%$ where $x_{{{\text{qNMR}}}} = \frac{{I_{{{\text{MC}}}} }}{{I_{{{\text{IS}}}} }} \cdot \frac{{N_{{{\text{IS}}}} }}{{N_{{{\text{MC}}}} }} \cdot \frac{{M_{{{\text{MC}}}} }}{{M_{{{\text{IS}}}} }} \cdot \frac{{m_{{{\text{IS}}}} }}{{m_{{{\text{MC}}}} }} \cdot P_{{{\text{IS}}}}$	(9)	*P*_qNMR_ is the purity of sample determined by qNMR; *x*_qNMR-Calc_ is the calculated purity from ^1^H NMR experiments; *I*_*I*S_ and *I*_MC_ are integrated area of standard and sample; *N*_IS_ and *N*_MC_ are numbers of spin of standard and sample; *M*_IS_ and *M*_MC_ are molar masses of standard and sample; *m*_IS_ and *m*_MC_ are weights of standard and sample; *P*_IS_ is the purity of internal standard
$\left( {\frac{{u(x_{{{\text{oi}}}} )}}{{x_{{{\text{oi}}}} }}} \right)^{2} = \sum {\left( {\frac{{u(A_{{{\text{oi}}}} )}}{{A_{{{\text{oi}}}} }}} \right)^{2} } + \left( {\frac{{u(A_{{{\text{tot}}}} )}}{{A_{{{\text{tot}}}} }}} \right)^{2}$	(10)	*u*(*x*_oi_) is the uncertainty of organic impurities determined by MB; *u(A*_oi_) is the uncertainty of the corrected chromatographic peak area of organic impurities. *u(A*_tot_) is the uncertainty of the corrected chromatographic peak area of all compounds
$u(x_{{{\text{qNMR}}}} ) = u(x_{{{\text{mo}}}} ) = u(x_{{{\text{re}}}} ){ = }u(x_{{{\text{sa}}}} ){\text{ = S/}}\sqrt {{10}}$	(11)	*u*(*x*_qNMR_) is the uncertainty of Sal A determined by qNMR; *u*(*x*_mo_), *u*(*x*_re_) and *u*(*x*_sa_) is the uncertainty of moisture, residual solvent and sulfated ash determined by MB, respectively; *S* is the standard deviation of relative experiments
$u\left( {{\text{MB}}} \right) = \sqrt {u^{2} \left( {x_{{{\text{oi}}}} } \right) + u^{2} \left( {x_{{{\text{mo}}}} } \right) + u^{2} \left( {x_{{{\text{sa}}}} } \right)}$	(12)	*u*(MB) is the uncertainty of purity determined by MB
$u\left( {{\text{qNMR}}} \right) = \sqrt {u^{2} \left( {x_{{{\text{qNMR}}}} } \right) + u^{2} \left( {x_{{{\text{mo}}}} } \right) + u^{2} \left( {x_{{{\text{sa}}}} } \right)}$	(13)	*u*(qNMR) is the uncertainty of purity determined by qNMR
$u_{{{\text{CRM}}}} = \sqrt {u(MB)^{2} + u({\text{q}}NMR)^{2} + u_{{\text{h}}}^{{2}} + u_{{{\text{sts}}}}^{{2}} + u_{{{\text{lts}}}}^{{2}} }$	(14)	*u*_CRM_ is the combined standard uncertainty of certified property value
$U_{{{\text{CRM}}}} = u_{{{\text{CRM}}}} k$	(15)	*U*_CRM_ is the expanded uncertainty of certified property value; *k* is the coverage factor

## Results and Discussions

### Structure Validation

The experimental data of structure analysis are shown below. ^1^H-NMR (CD_3_OD) *δ* ppm: 2.901 (dd, *J* = 8.8 Hz, 14.4 Hz), 3.019 (dd, *J* = 4.0 Hz, 14.4 Hz), 5.113 (dd, *J* = 4.0 Hz, 8.8 Hz), 5.580 (d, *J* = 16.0 Hz), 6.233 (d, *J* = 16.0 Hz), 6.480 (dd, *J* = 8.0 Hz, 1.6 Hz), 6.582 (d, *J* = 8.0 Hz), 6.654 (d, *J* = 1.6 Hz), 6.687 (d, *J* = 8.0 Hz), 6.696 (d, *J* = 8.0 Hz), 6.823 (dd, *J* = 8.0 Hz, 1.6 Hz), 6.986 (d, *J* = 1.6 Hz), 7.059 (d, *J* = 8.0 Hz), 7.085 (d, *J* = 16.0 Hz), 8.003 (d, *J* = 16.0 Hz). ^13^C-NMR (CD_3_OD) *δ* ppm: 37.544 (A7), 74.745 (A8), 113.907 (C2), 114.753 (B5), 115.571 (B5), 116.285 (A5), 116.432 (C5), 117.353 (A2), 120.061 (B6), 120.434 (C6), 120.640 (C8), 121.946 (A6), 126.083 (B1), 128.346 (B2), 129.294 (A1), 131.383 (C1), 137.845 (C7), 144.400 (B3), 145.202 (A4), 146.119 (A3), 146.460 (C3), 146.746 (C4), 147.345 (B7), 148.250 (B4), 168.620 (B9), 173.570 (A9). MS (m/z): 517 [M + Na]^+^. IR (KBr pellets): 3375, 1691, 1602, 1519, 1491, 1444, 1359, 1284, 1252, 1190, 1112, 1070, 1041, 969, 935, 864, 808, 757, 721, 627, 590, 488, 457 cm^−1^. UV (H_2_O) λ_max_: 286.0 nm. $\left[ \alpha \right]_{D}^{20} = 25.67$(0.5 mg/mL, 50% ethanol water). The data obtained were mainly identical to the data reported in References [1, 16, 17]; thus, the compound was identified as Sal A.

### Homogeneity and Stability Studies

**Fig. 1:**
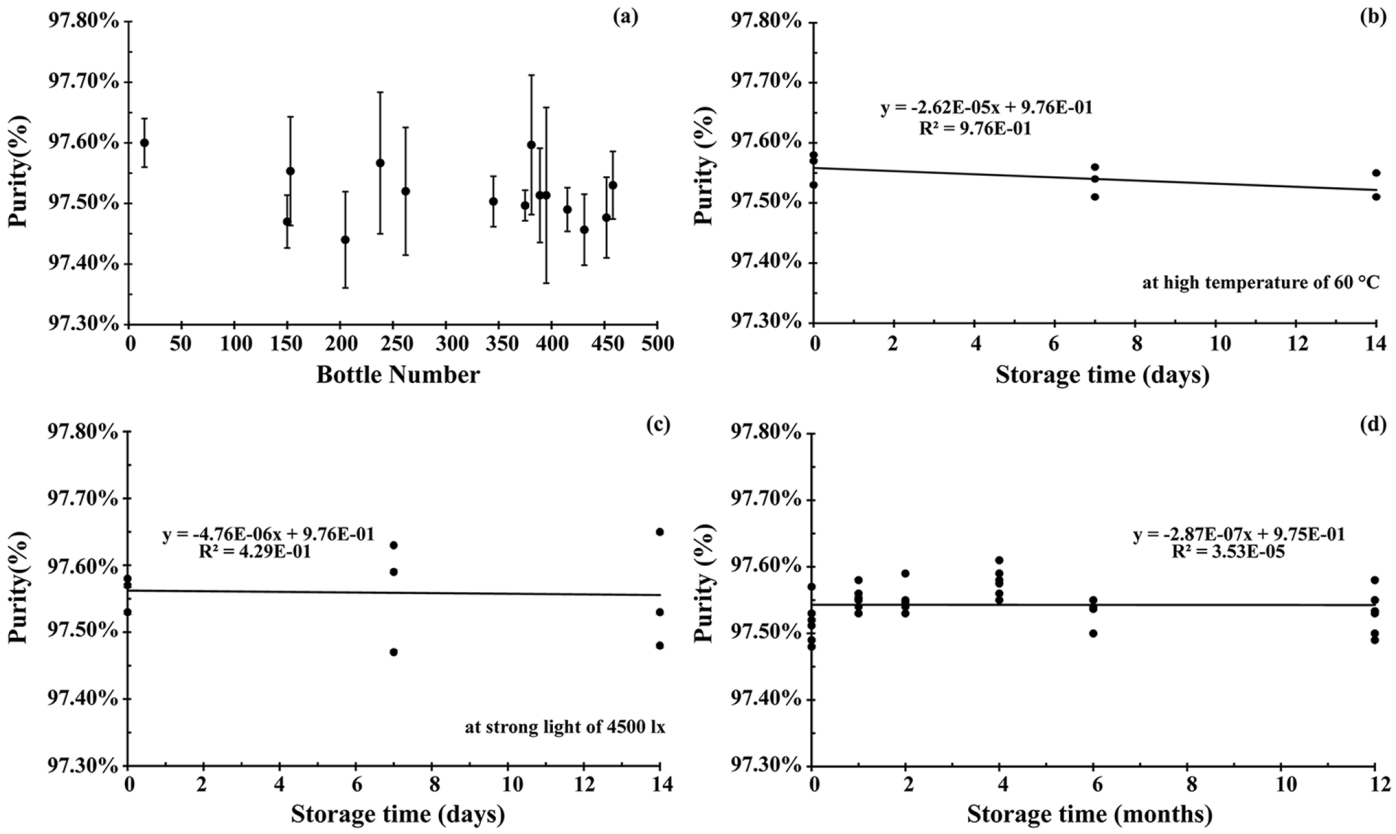
Homogeneity and stability assessments of CHA candidate CRM. **a** Homogeneity results of the 15 randomly picked bottles (mean ± standard deviation from 3 replicate measurements). Short-term stability results of the 3 randomly picked bottles for each of the time points (0–14 days): **b** high temperature, 60 °C, **c** strong light, 4500 lx. Long-term stability results of the 6 randomly picked bottles for each of the time points (0–12 months): **d** room temperature, 20 °C. The regression lines with the slope and intercept are indicated

A one-way analysis of variance (ANOVA, *F*-test) was used to evaluate the homogeneity of the Sal A CRM. The mean square between bottles (*MS*_*between*_) was larger than the mean square within bottles (*MS*_*within*_), which meant that the method has good repeatability. The ratio of mean square (*F*) was smaller than the critical value (*F*_*crit*_), which meant that the homogeneity of Sal A CRM was good. In this case, Eq. (1) was used to calculate *u*_H_. Table 3 shows the results of the homogeneity study of Sal A CRM and no trends were detected. Thus, the homogeneity of the CRM is suitable for the intended use (Fig. 1a). The *t*-test was used to evaluate the short-term stability. According to Eq. (4), the calculated values of *t* on different conditions were all less than the critical value (*t*_*crit*_) from the *t* table of critical values, thereby indicating that the Sal A CRM has good short-term stability and this observation indicated that the CRMs were sufficiently stable for dispatch under the transport conditions (Fig. 1b, c). Linear regression analysis was used to evaluate the long-term stability. The slope and standard deviation of the data points obtained in the long-term stability studies were calculated using Eqs. (2) and (3). The absolute values of the slopes were smaller to the product of time (12/month) and slope uncertainty, thereby indicating that the Sal A CRM has good long-term stability (Fig. 1d). The result showed that the Sal A CRM would maintain its stability for up to 1 year under the conditions in this study. The uncertainties of short- and long-term stabilities were evaluated by using Eqs. (5) and (7). Table 3 shows the results of short- and long-term stabilities of Sal A CRM.

**Table 3:** Certification results of Sal A candidate CRM

	Parameters	Results
Homogeneity studies	*F*	1.05
	*F*_crit_	2.04
	*MS*_between_	6.84 × 10^–7^
	*MS*_within_	6.50 × 10^–7^
	*u*_H_	1.06 × 10^–4^
Stability studies	*S*_*(b)* (temp)_	6.63 × 10^–5^
	*S*_*(b)* (photo)_	7.30 × 10^–5^
	*S*_*(b)*_	6.28 × 10^–5^
	*u*_sts(temp)_	9.29 × 10^–4^
	*u*_sts(photo)_	1.02 × 10^–3^
	*u*_sts_	1.38 × 10^–3^
	*u*_lts_	7.54 × 10^–4^
Purity determination	HPLC (uncorrected)	97.43%
	HPLC (corrected)	95.67%
	Organic impurity	4.33%
	^1^H NMR	95.79%
	Moisture	0.52%
	Residual solvent	0.32%
	Macroreticular resin residues	Undetected
	Polyamide resin residues	Undetected
	Inorganic impurities	0.21%
		23.42 μg/g (ICP-MS)
	MB method	94.62%
	qNMR method	94.74%
	Raw material	82.32%
Uncertainty estimation	*u* (MB)	0.15%
	*u* (qNMR)	0.08%
	*u*_CRM_	0.23%
	*U*_CRM_	0.47%
Results of CRM analysis	*k* = 2*, P* = 0.95	94.7% ± 0.5%

**Fig. 2:**
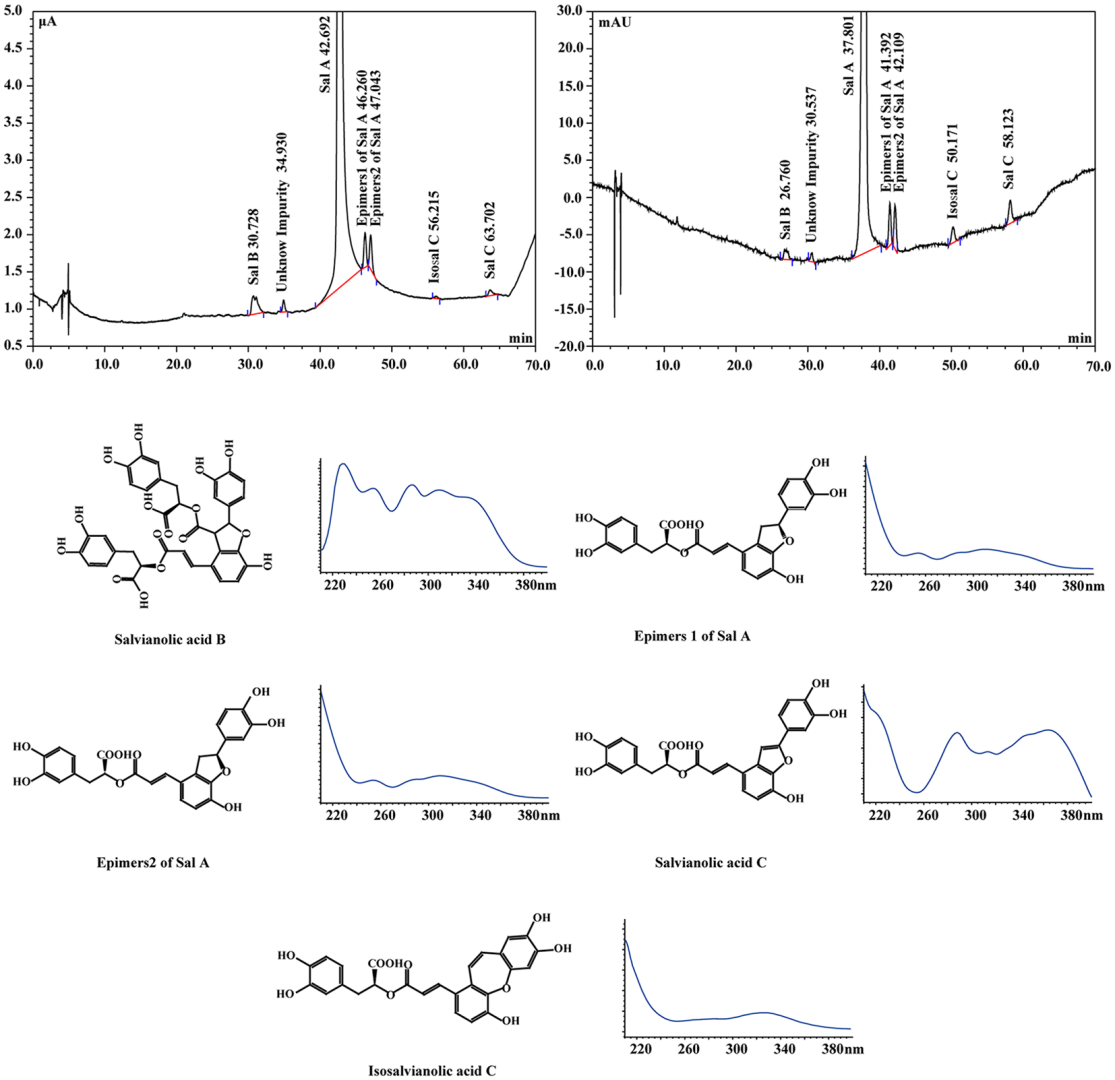
Typical ECD and DAD chromatograms and ultraviolet absorption spectrum of Sal A and impurities

Six trace impurities in Sal A CRM were determined by HPLC/MS. Preparative high performance liquid chromatography (PHPLC) was used to separate and prepare these impurities, and their structures were confirmed by MS and NMR (Fig. 2) and related experimental information are listed in the supplementary information.

Calibration factors of impurities were determined by HPLC. In this paper, the relative purity of Sal A was determined by using area normalization method with correction factor.

**Fig. 3:**
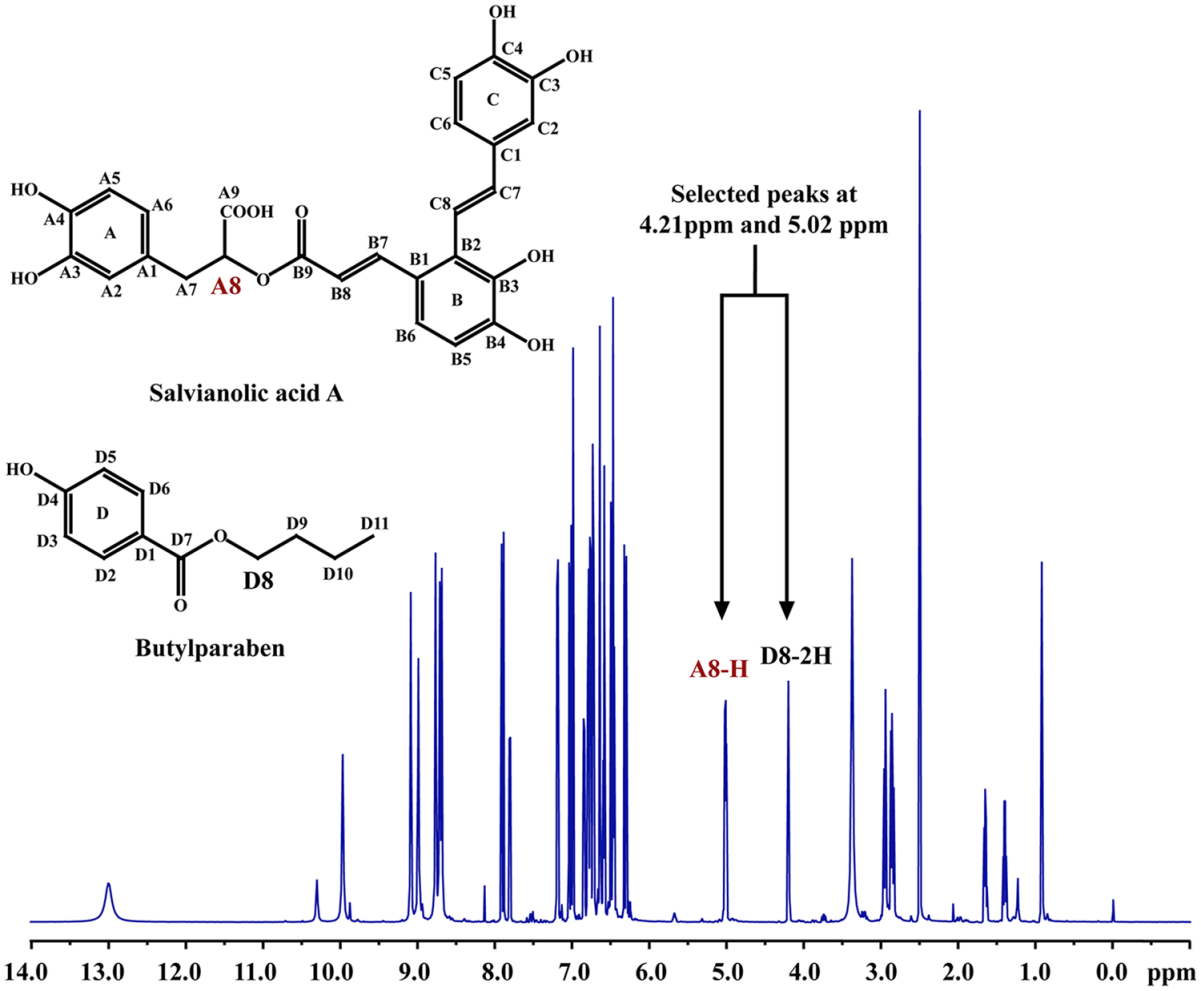
Quantitative ^1^H-NMR spectrum of Sal A with BPB internal standard

Organic impurities, inorganic impurities, water, solvent residues, and toxic residues of different resins were carefully determined, and the results are listed in Table 3. Mass balance method requires the determination of the contents of various impurities first, including non-volatile impurities (organic impurities), moisture, residual solvent, and inorganic impurities. In recent years, some researchers use ICP-MS to determine the content of inorganic impurities, so in this research, we also use this method to analyze the candidate CRM. The results showed that result obtained form ICP-MS was far less than that on ignition residue method, in view of suitability of methods for this CRM, this study finally used the latter result to calculate the content of inorganic impurities. The content of the sample can be obtained by subtracting the content of impurities from the total content (100%). Therefore, the purity of Sal A was 94.62% and 94.74% as determined by MB method and qNMR method (Fig. 3), respectively.

The results determined through MB were compared with those acquired through qNMR via *t*-test, and consistent results were obtained. Hence, the purity of the candidate CRM can be expressed by the arithmetic mean of the results from these methods. The findings of characterization and uncertainty evaluation are shown in Table 3.

The combined standard uncertainties (*u*_CRM_) and expanded uncertainties (*U*_CRM_) for the CRMs were calculated using Eqs. (13) and (14), respectively. Table 3 shows the results of *u*_CRM_ and *U*_CRM_ estimations.

## Conclusion

In summary, high-purity Sal A was obtained by using sephadex chromatography and preparative chromatography. Moreover, according to ISO Guides, Sal A CRM was developed and achieved accurate purity value and uncertainty estimation. Sal A CRM can be applied in the development of new drugs, quality control of raw materials, preparations, health food and validation of analysis methods.

## Experimental

### Materials

Sal A raw material with a purity of 82.32% as determined by HPLC, was provided by the National Center for Pharmaceutical Screening of China. Butylparaaben (CAS: 94-26-8) was purchased from Sigma-Aldrich.

### Instruments

HPLC measurements were performed using an Agilent 1260 liquid chromatographic system with a diode array detector (Agilent Technologies, Inc., USA) and a Thermo Scientific Dionex UltiMate 3000 RS electrochemical detector. An Agilent 7890A GC system (Agilent Technologies, Inc., USA) was used to determine the residual solvents. NMR was performed on a 600 MHz VNS NMR spectrometer (Varian, Palo Alto, CA, USA) with DMSO-*d*_6_ as solvent at 25 °C. High resolution MS and HPLC/MS experiments were carried out on an AccuTOF CS JMS-T100CS mass spectrometer (JEOL Ltd., Tokyo, Japan). ICP-MS measurements were performed on an inductively coupled plasma mass spectroscopy "NexIon 350X" of PerkinElmer (ICP-MS, Waltham, MA, USA). Sample was digested using a closed-vessel microwave instrument "CEM MARs Xpress" (CA, USA) before analysis. Preparative isolation work was conducted on a Shimadzu LC-6A series preparative HPLC system (Kyoto, Japan) equipped with a manual injector, binary pump, and photodiode array detector. The moisture of substances was determined by a Mettler-Toledo DL 39 Karl Fischer coulometric titrator (Mettler-Toledo Inc., Switzerland). A SX25-01 muffle furnace (Shanghai Shuli, Inc., China) was used for sulfated ash measurements.

### Preparation and Purification

About 60 g Sal A raw material was dissolved in 150 mL methanol, and the solution was purified through Sephadex LH-20 column chromatography by using methanol with isocratic elution. The eluant containing Sal A (> 95% purity, as determined by HPLC) was collected and converted to solid by rotary evaporation. Afterward, the solid was dissolved with a mixture of acetonitrile and 0.1% formic acid aqueous solution with a volume ratio of 3:7, and the Sal A concentration in the solution was about 150 mg/mL. The follow-up purification was performed by preparing liquid chromatography, and the chromatographic condition was done as follows: The preparative column used was a C18 column (20 mm × 250 mm, 5 μm), and the column temperature was 25 °C. A mixture of the mobile phase of 0.1% (v/v) formic acid aqueous solution and acetonitrile at a volume ratio of 70:30 (v/v) was used as the mobile phase for sample preparation. The injection volume was 1 mL. The flow rate was 5 mL/min and the constituents were monitored at 286 nm. The Sal A and impurity fractions were collected separately from several injections and pooled separately. Afterward, the pooled fractions were concentrated by using a rotary evaporator under high vacuum. The final products were obtained through a freeze-dryer under the condition of −40 °C and 1 Pa. All samples were kept in a dry place at approximately 4 °C.

### Structure Validation

Nuclear magnetic resonance (NMR) spectroscopy, mass spectrometry (MS), infrared (IR) spectrometry, ultraviolet (UV) spectrophotometry, and optical rotation (OR) were used for the structural analysis of Sal A.

### Homogeneity and Stability Studies

Homogeneity and stability are the main characteristics of CRM. Homogeneity is a condition of having a uniform structure or composition with respect to one or more specified properties. Stability is the ability of CRM to maintain a stated property value within specified limits for a specified period of time when stored under specified conditions. Stability is divided into long-term stability (storage conditions) and short-term stability (transport conditions).

A total of 15 candidate CRMs were randomly selected to perform the homogeneity study using HPLC, and the measurement data were assessed by analysis of variance (ANOVA). Two conditions were set, namely, high temperature (60 °C) and strong illumination (4500 lx ± 500 lx) for the short-term stability. A total of 6 randomly selected candidate CRMs were placed in each condition separately for 14 days. Every 7 days, 3 of candidates were analyzed by HPLC. The *t*-test was used to evaluate the short-term stability. A total of 36 candidate CRMs were randomly selected to assess long-term stability, and 6 of them were analyzed by HPLC at 25 °C on 0, 1, 2, 4, 6, and 12 months. Linear regression analysis was employed to evaluate long-term stability.

### Organic Impurities Analysis

HPLC–DAD tandem ECD and HPLC–DAD tandem MS methods were used for organic impurity analysis. A total of 6 impurities were found and five of their structures were validated by using MS and NMR. Area normalization method with correction factor as determined by HPLC was employed to quantify the contents of the impurities.

Operational conditions of HPLC included the following: flow rate at 1.0 mL/min, injection volume at 10 μL, column temperature at 25 °C, the wavelength of DAD ranged from 200 to 400 nm, and detection was set to 286 nm. The mobile phase consisted of 0.5% (v/v) acetic acid solution (A) and acetonitrile (B). A gradient program was used as follows: elution starting with 20% B, increased to 28% after 15 min, and then increased to 45% at the 70th min. The sample solution concentration was 0.5 mg/mL.

### NMR Spectrometer

Due to the advantages of its feasibility, high accuracy, simultaneous qualitative and quantitative analysis, as well as the content determination without its own reference substance, qNMR has been widely used as a method for determination of the mass fraction of a pure compound, compared with other techniques, such as HPLC or GC. Many studies have reported its applications for the purity assessment of pure substances and quality control in pharmaceutical area [17]. Nowadays, qNMR has been adopted as the standard method by the pharmacopeias of many countries including the USA, UK, EU, JP and CHN, etc. [18]. In this work, butylparaaben (BPB) was chosen as internal standard for qNMR analysis. Sal A (appr. 20 mg) and benzoic acid (appr. 2 mg) were dissolved in DMSO‑*d*_6_ (0.5 mL), and the solution was pipetted into the NMR tube and then covered with a cap. The ^1^H NMR spectra was performed with a proton frequency of 600.23 MHz and a temperature of 298 K. Each experiment consisted of 16 scans with a spectral width of 12,019.2 Hz and a recycle delay of 15 s per scan.

### Solvent Residue Analysis

Solvents of methanol, ethanol, acetone, and ethyl acetate were used in the preparation and purification of Sal A. Therefore, headspace gas-chromatography (GC–HS) was used for solvent residue analysis in this paper.

Shimadzu GC-2014 gas chromatograph (FDI detector) was used with a headspace sampler and SE-54 capillary column (30 m × 0.32 mm × 0.25 μm). Injection port temperature was set to 180 °C; split ratio was 1:20; column temperature was maintained at 40 °C for 6 min; heat was set to 180 °C at a rate of 15 °C/min; detector temperature was set to 250 °C; carrier gas was nitrogen with a constant speed of 1.7 mL/min; heating furnace temperature was maintained at 85 °C for 30 min; quantitative tube temperature was set to 90 °C; transfer tube temperature was set to 95 °C; and injection volume was 1 mL.

### Macroreticular Resin Residues Analysis

In the process of preparation, we used macroreticular resin D101, which may introduce some toxic residues, mainly including benzene, toluene, xylene, styrene, and alkane. Therefore, these toxic residues should be identified and quantified. Gas-chromatography (GC) method was used for macroreticular resin residue analysis in this paper.

Agilent 7890A gas chromatograph (FDI detector) was used with a headspace sampler. Agilent GC/MS Chemstation with DB-FFAP capillary column (30 m × 0.320 mm × 0.25 μm) was used. Injection port temperature was 250 °C; detector temperature was set to 280 °C; column temperature was maintained at 35 °C for 10 min; heat was set to 45 °C at a rate of 3 °C/min, and then maintained for 5 min; heat was increased to 220 °C at a rate of 30 °C/min and maintained for 5 min; carrier gas was helium with a constant speed of 1.0 mL/min; and split ratio was set to 20:1. Heating furnace temperature was maintained at 90 °C for 30 min; quantitative tube temperature was set to 100 °C; transfer tube temperature was set to 120 °C; and the injection volume was 1 mL.

### Polyamide Resin Residues Analysis

Some monomers and oligomers of caprolactam were not involved in the polymerization reaction of plyamide, which could be eluted into products during column chromatography. Caprolactam is an irritant and a mildly toxic substance that could cause spasm. Therefore, its residue should be identified and its content should be quantified. Caprolactam oligomers have no ultraviolet absorption; thus, they are hydrolyzed to aminocaproic acid by acid hydrolysis for approximately 14 h, and then determined by HPLC. With this method, caprolactam and its oligomers can be quantified.

The operational condition of HPLC included the following: flow rate at 0.5 mL/min, injection volume at 10 μL, Waters Atlantis® T3 column (4.6 × 250 mm, 5 μm) at 30 °C, and detection wavelength 210 nm. The mobile phase consisted of water (A) and methanol (B). A gradient program was used as follows: elution starting with 10% B, and increased to 20% after 15 min.

### Moisture Content and Sulphated Ash Analysis

Moisture was determined by using the Karl-Fischer method and the inorganic impurities were acquired through the routine method of residue on ignition and ICP-MS method.

## Electronic supplementary material

Below is the link to the electronic supplementary material.

Electronic supplementary material 1 (DOC 590 kb)
