# Supplementary material for: Preparation and Certification of a New Salvianolic Acid A Reference Material for Food and Drug Research
Source: Nat Prod Bioprospect. 2020 Apr 18;10(2):67–75. doi: 10.1007/s13659-020-00236-2 (PMC7176770; doi:10.1007/s13659-020-00236-2)
Supplement: Supplementary file 1 — Electronic supplementary material 1 (DOC 590 kb) [file 13659_2020_236_MOESM1_ESM.doc]

**
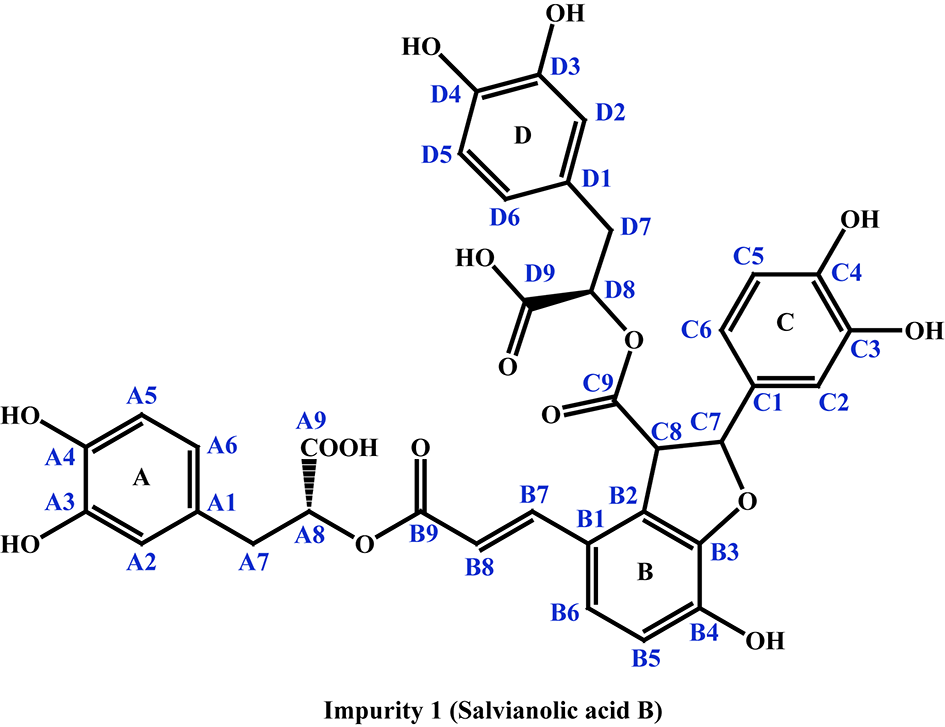
**

**Fig 1. The chemical structure of the impurity 1 (Sal B).**

The MS spectrum of impurity 1 exhibited a quasi-molecular [M+Na]^+^ of 741.1423 in positive ion mode. Impurity 1 was Sal B for their similar MS data and retention time as the reference. The structure of impurity 1 was shown in Figure 1.

**
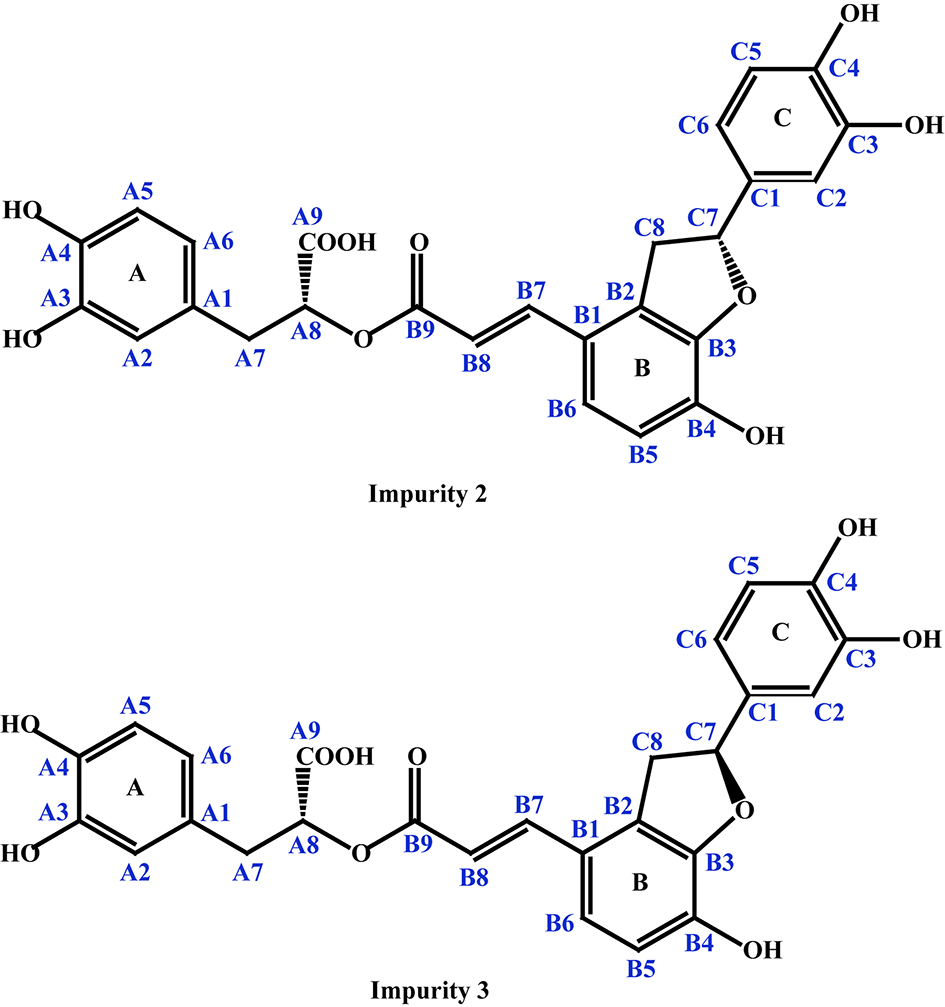
**

**Fig 2. The chemical structure of the impurity 2 and 3.**

The MS spectrum of impurity 2 and 3 exhibited quasi-molecular [M+Na]^+^ of 517.11258 in positive ion mode. Impurity 2 and 3 were a pair of epimers of Sal A for their similar NMR data and MS data. The structure of impurity 2 and 3 were shown in Figure 2.

**Table 1**

**NMR Data for impurity 2 and 3**

| **position** | **experimental**  **value of ^13^C NMR** | **literature value of ^13^C NMR** | **experimental**  **value of ^1^H NMR** | **literature value**  **of ^1^H NMR** |
| --- | --- | --- | --- | --- |
| **A1** | 129.564 | 129.5 | - | - |
| **A2** | 117.532 | 117.7 | 6.724(br) | 6.73(d,J=2.5Hz) |
| **A3** | 145.952 | 146.3 | - | - |
| **A4** | 144.875 | 145.4 | - | - |
| **A5** | 116.776 | 116.4 | 6.614(d,J=8.4Hz) | 6.67(d,J=8.0Hz) |
| **A6** | 121.752 | 121.9 | 6.548(br) | 6.60(d,J=8.0Hz) |
| **A7** | 38.494 | 38.1 | 3.031(m),2.902(m) | 3.09(m),2.99(m) |
| **A8** | 76.902 | 75.1 | 5.055(br) | 5.17(dd,J=4.0Hz,8.0Hz) |
| **A9** | - | 172.4 | - | - |
| **B1** | 123.900 | 123.9 | - | - |
| **B2** | 130.583 | 129.9 | - | - |
| **B3** | 148.255 | 148.5 | - | - |
| **B4** | 144.470 | 145.1 | - | - |
| **B5** | 117.001 | 117.2 | 6.645(d,J=8.4Hz) | 6.72(d,J=8.3) |
| **B6** | 122.962 | 123.1 | 6.945(d,J=8.4Hz) | 7.06(d,J=8.3) |
| **B7** | 144.689 | 145.3 | 7.517(d,J=16.2Hz) | 7.62(d,J=16.0) |
| **B8** | 116.255 | 116.1 | 6.171(d,J=16.2Hz) | 6.25(d,J=16.0) |
| **B9** | 168.861 | 168.5 | - | - |
| **C1** | 134.704 | 134.8 | - | - |
| **C2** | 114.084 | 114.2 | 6.815(s) | 6.87(d,J=2.0) |
| **C3** | 146.408 | 146.6 | - | - |
| **C4** | 146.435 | 146.7 | - | - |
| **C5** | 116.823 | 116.4 | 6.724(br) | 6.75(d,J=9.2) |
| **C6** | 118.886 | 119.0 | 6.724(br) | 6.78(d,J=9.2) |
| **C7** | 86.162 | 86.3 | 5.621(m) | 5.71(m) |
| **C8** | 39.377 | 39.5 | 3.631(m),3.165(m) | 3.73(m),3.26(m) |

**
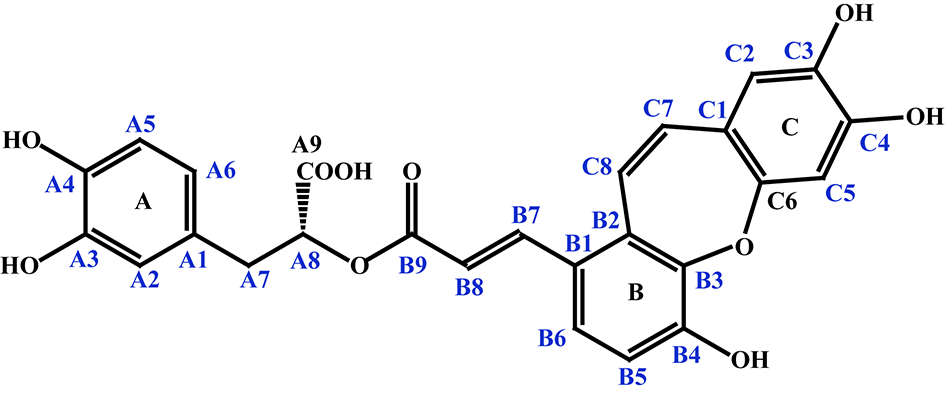
**

**Fig 3. The chemical structure of the impurity 4 (Isosal C).**

The MS spectrum of impurity 4 exhibited a quasi-molecular [M+Na]^+^ of 515.09672 in positive ion mode. Impurity 4 was Isosal C for their similar NMR data and MS data. The structure of impurity 4 was shown in Figure 3.

**Table 2**

**NMR Data for impurity 4**

| **position** | **experimental value of ^13^C NMR** | **literature value of ^13^C NMR** | **experimental value of ^1^H NMR** | **literature value**  **of ^1^H NMR** |
| --- | --- | --- | --- | --- |
| **A1** | 128.728 | 126.7 | - | - |
| **A2** | 116.612 | 116.7 | 6.65(br) | 6.64(d, J=1.8Hz) |
| **A3** | 144.945 | 144.2 | - | - |
| **A4** | 143.759 | 145.5 | - | - |
| **A5** | 115.387 | 115.5 | 6.60(br) | 6.65(d,J=8.4Hz) |
| **A6** | 119.895 | 120.2 | 6.51(d,J=8.4Hz) | 6.53(dd,J=8.4Hz,1.8Hz) |
| **A7** | 36.690 | 36.2 | 2.83,3.01(br) | 3.00(dd,J=14.4Hz,4.8Hz)，  2.96(dd,J=14.4Hz,7.8Hz) |
| **A8** | 74.679 | 72.9 | 4.93(br) | 5.14(dd,J=7.8Hz,2.7Hz) |
| **A9** | 171.811 | 169.9 |  |  |
| **B1** | 122.641 | 122.5 | - | - |
| **B2** | 130.942 | 131.3 | - | - |
| **B3** | 145.112 | 145.2 | - | - |
| **B4** | 151.042 | 151.3 | - | - |
| **B5** | 116.821 | 116.9 | 6.9(d,J=8.4Hz) | 6.89(d,J=8.4Hz) |
| **B6** | 124.069 | 124.4 | 7.4(d,J=8.4Hz) | 7.47(d,J=8.4Hz) |
| **B7** | 140.698 | 142.6 | 7.74(d,J=15.6Hz) | 7.82(d,J=15.9Hz) |
| **B8** | 117.164 | 115.8 | 6.26(d,J=15.6Hz) | 6.34(d,J=15.9Hz) |
| **B9** | 165.747 | 165.8 | - | - |
| **C1** | 120.989 | 121.1 | - | - |
| **C2** | 114.640 | 114.6 | 6.63(br) | 6.63(s) |
| **C3** | 147.339 | 147.3 | - | - |
| **C4** | 150.039 | 150.1 | - | - |
| **C5** | 108.961 | 108.9 | 6.82(br) | 6.84(s) |
| **C6** | 142.621 | 142.1 | - | - |
| **C7** | 131.766 | 132.1 | 6.82(br) | 6.87(1H,d,11.4) |
| **C8** | 123.283 | 123.3 | 6.82(br) | 6.84(1H,d,11.4) |

***
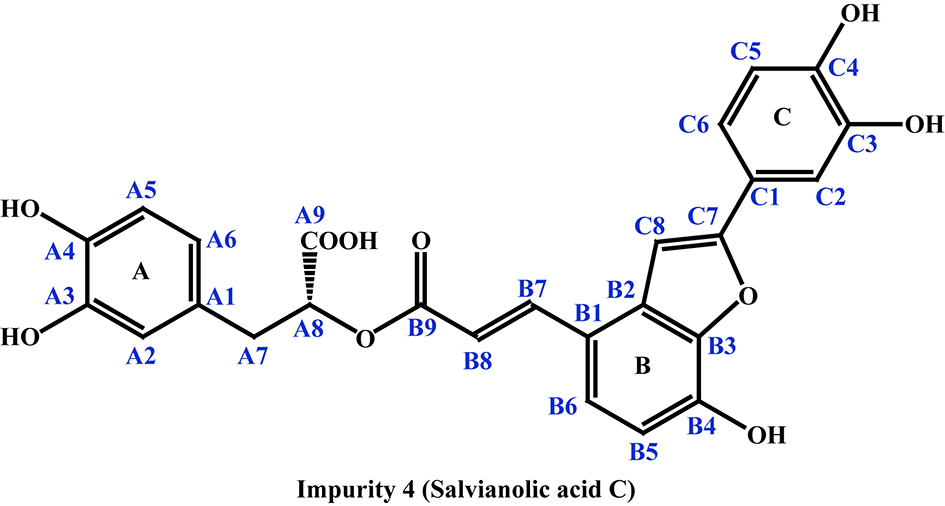
***

**Fig 4. The chemical structure of the impurity 5 (Sal C).**

The MS spectrum of impurity 4 exhibited a quasi-molecular [M+Na]^+^ of 515.09672 in positive ion mode. Impurity 5 was Sal C for their similar NMR data and MS data. The structure of impurity 5 was shown in Figure 4.

**Table 3**

**NMR Data for impurity 5**

| **position** | **experimental value of ^13^C NMR** | **literature value of ^13^C NMR** | **experimental value of ^1^H NMR** | **literature value**  **of ^1^H NMR** |
| --- | --- | --- | --- | --- |
| **A1** | 129.317 | 129.8 | - | - |
| **A2** | 117.653 | 117.7 | 6.792(d,J=1.8Hz) | 6.82(d,J=2.0Hz) |
| **A3** | 145.329 | 145.2 | - | - |
| **A4** | 144.328 | 144.1 | - | - |
| **A5** | 116.328 | 116.6 | 6.717(d,J=7.8Hz) | 6.69(d,J=8.1Hz) |
| **A6** | 121.835 | 121.6 | 6.651(dd,J=7.8Hz,1.8Hz) | 6.62(dd,J=8.1Hz,2.0Hz) |
| **A7** | 37.976 | 38.0 | 3.132(dd,J=14.4Hz,4.2Hz),  3.052(dd,J=14.4Hz,8.4Hz) | 3.07(dd,J=14.2Hz,3.9Hz),  2.96(d,J=14.2,8.8Hz) |
| **A8** | 74.658 | 74.7 | 5.245(dd,J=14.4Hz,8.4Hz) | 5.16(dd,J=8.8Hz,3.9Hz) |
| **A9** | 173.607 | - | - | - |
| **B1** | 123.331 | 123.2 | - | - |
| **B2** | 132.713 | 132.8 | - | - |
| **B3** | 146.220 | 146.1 | - | - |
| **B4** | 148.060 | 148.0 | - | - |
| **B5** | 111.771 | 112.1 | 6.733(d,J=8.4Hz) | 6.77(d,J=8.3Hz) |
| **B6** | 118.772 | 118.8 | 7.379(d,J=8.4Hz) | 7.37(d,J=8.3Hz) |
| **B7** | 145.204 | 144.5 | 7.930(d,J=14.4Hz) | 7.85(d,J=16.1Hz) |
| **B8** | 116.750 | 116.8 | 6.464(d,J=14.4Hz) | 6.45(d,J=16.1Hz) |
| **B9** | 168.678 | 167.8 | - | - |
| **C1** | 119.588 | 119.6 | - | - |
| **C2** | 114.874 | 115.7 | 7.396(d,J=1.8Hz) | 7.45(d,J=2.2Hz) |
| **C3** | 146.025 | 145.9 | - | - |
| **C4** | 146.747 | 146.7 | - | - |
| **C5** | 113.402 | 113.4 | 6.877(d,J=8.4Hz) | 6.88(d,J=8.3Hz) |
| **C6** | 126.292 | 126.6 | 7.365(d,J=8.4Hz) | 7.35(dd,J=8.3Hz,2.2Hz) |
| **C7** | 159.440 | 159.2 | - | - |
| **C8** | 99.251 | 99.8 | 7.209(s) | 7.36(s) |

***1. Detection limit, Quantitation limit and Linearity of 16 water-soluble ingredients in Danshen***

**Table 4**

LOD, LOQ and Linearity of 16 water-soluble ingredients in Danshen

| **No.** | **Compounds** | **LOD**  **(μg/ml)** | **LOQ**  **(μg/ml)** | **Linearity** |
| --- | --- | --- | --- | --- |
| 1 | Danshensu | 2.0 | 5.0 | y=21.1984x-236.6540, R^2^=0.9996 |
| 2 | 3,4-Dihydroxybenzoic acid | 0.3 | 1.0 | y=16.5944x+33.1057, R^2^=1.0000 |
| 3 | Protocatechualdehyde | 0.2 | 1.0 | y=38.4028x-291.3181, R^2^=0.9993 |
| 4 | Caffeic acid | 0.2 | 0.6 | y=39.2172x-6.4005, R^2^=0.9997 |
| 5 | Ferulic Acid | 0.2 | 0.6 | y=36.7740x+93.8444, R^2^=0.9998 |
| 6 | 3-Hydroxy-4-methoxycinnamic acid | 0.2 | 0.6 | y=41.3653x+1.3949, R^2^=0.9999 |
| 7 | Rosmarinic acid | 2.0 | 5.0 | y=21.1984x-236.6540, R^2^=0.9996 |
| 8 | Lithospermic acid | 1.0 | 3.0 | y=14.3189x-64.9018, R^2^=0.9999 |
| 9 | Salvianolic acid B | 1.0 | 5.0 | y=11.3730x-45.8375, R^2^=0.9999 |
| 10 | Unknow impurity I | 0.5 | 2.0 | y=16.100x-64.8483, R^2^=0.9998 |
| 11 | Unknow impurity II | 0.5 | 2.0 | y=16.100x-64.8483, R^2^=0.9998 |
| 12 | 9'''-Methyllithospermate B | 2.0 | 5.0 | y=11.2864x-36.7588, R^2^=0.9999 |
| 13 | Isosalvianolic acid C | 0.2 | 0.5 | y=17.0603x+27.4975, R^2^=0.9996 |
| 14 | Salvianolic acid C | 1.0 | 3.0 | y=26.0022x-18.345, R^2^=0.9999 |
| 15 | Methyl salvionolate A | 0.3 | 1.0 | y=30.6839x-154.2610, R^2^=0.9999 |
| 16 | Dimethyl lithospermate B | 2.0 | 5.0 | y=11.5236x+34.0417, R^2^=0.9999 |

**Table 5**

***Determination results of the correction factors (n=3)***

| **Compounds** | **Slope of Regression Equation** | ***f*** |
| --- | --- | --- |
| Salvianolic acid A | 32.00 | 1.00 |
| Salvianolic acid B | 11.37 | 0.35 |
| Impurity 2 | 16.11 | 0.50 |
| Impurity 3 | 17.91 | 0.56 |
| Isosalvianolic acid C | 17.06 | 0.53 |
| Salvianolic acid C | 26.00 | 0.81 |

***2. Homogeneity study of Sal A candidate CRM***

**Table 6**

**The results of homogeneity study of Sal A candidate CRM**

| **Random No.** | **Purity (%)** | | |
| --- | --- | --- | --- |
|  | **1** | **2** | **3** |
| **452** | 97.46 | 97.55 | 97.42 |
| **431** | 97.39 | 97.48 | 97.50 |
| **375** | 97.47 | 97.50 | 97.52 |
| **415** | 97.45 | 97.50 | 97.52 |
| **345** | 97.47 | 97.49 | 97.55 |
| **238** | 97.67 | 97.44 | 97.59 |
| **389** | 97.45 | 97.60 | 97.49 |
| **458** | 97.58 | 97.54 | 97.47 |
| **150** | 97.44 | 97.45 | 97.52 |
| **262** | 97.62 | 97.41 | 97.53 |
| **395** | 97.37 | 97.66 | 97.51 |
| **381** | 97.48 | 97.71 | 97.60 |
| **15** | 97.64 | 97.56 | 97.60 |
| **205** | 97.47 | 97.50 | 97.35 |
| **153** | 97.45 | 97.61 | 97.60 |

**Table 7**

**The results of ANOVA**

|  | **SS** | **df** | **MS** | **F** | **P-value** | **F crit** |
| --- | --- | --- | --- | --- | --- | --- |
| Between | 9.57×10^-6^ | 14 | 6.84×10^-7^ | 1.05 | 0.43 | 2.04 |
| Within | 1.95×10^-5^ | 30 | 6.50×10^-7^ |  |  |  |
| Total | 2.91×10^-5^ | 44 |  |  |  |  |

$$u_{h}=\sqrt{\left(MS_{between}-MS_{within}\right)/n}\text{=}\sqrt{\left(6.84\times10^{\text{-7}}\text{-6.50}\times10^{\text{-7}}\right)/3}\text{=1.06}\times10^{\text{-4}}$$

***3. Stability studies***

***3.1 Short-term stability of Sal A candidate CRM***

**Table 8**

**The results of short-term stability of Sal A candidate CRM**

| **Conditions** | **Time**  **(day)** | **Purity (%)** | | | | ***S*** |
| --- | --- | --- | --- | --- | --- | --- |
|  |  | **1** | **2** | **3** | **Mean** |  |
| high temperature  (60 °C) | 0 | 97.53 | 97.58 | 97.57 | 97.56 | 0.000265 |
|  | 7 | 97.63 | 97.59 | 97.47 | 97.56 | 0.000833 |
|  | 14 | 97.53 | 97.48 | 97.65 | 97.55 | 0.000874 |
| high illumination  (4500 lx) | 0 | 97.53 | 97.58 | 97.57 | 97.56 | 0.000265 |
|  | 7 | 97.51 | 97.54 | 97.56 | 97.54 | 0.000252 |
|  | 14 | 97.51 | 97.55 | 97.51 | 97.52 | 0.000231 |

**Table 9**

**The results of uncertainty evaluation of short-term stability**

| **Conditions** | **Linear equation** | ***S*** | ***u_sts_*(Conditions)** | ***u_sts_*** |
| --- | --- | --- | --- | --- |
| high temperature | y = -4.76×10^-6^x + 9.76×10^-1^ | 6.57×10^-4^ | 9.29×10^-4^ | 1.38×10^-3^ |
| high illumination | y = -2.62×10^-5^x + 9.76×10^-1^ | 7.23×10^-4^ | 1.02×10^-3^ |

***3.2 Long-term stability of Sal A candidate CRM***

**Table 10**

***The results of long-term stability of Sal A candidate CRM***

| **Time**  **(month)** | **Purity (%)** | | | | | |  | ***S*** |
| --- | --- | --- | --- | --- | --- | --- | --- | --- |
|  | **1** | **2** | **3** | **4** | **5** | **6** | **Mean** |  |
| **0** | 97.53 | 97.48 | 97.57 | 97.48 | 97.52 | 97.49 | 97.51 | 0.00035 |
| **1** | 97.55 | 97.58 | 97.53 | 97.56 | 97.56 | 97.54 | 97.55 | 0.00018 |
| **2** | 97.54 | 97.59 | 97.53 | 97.54 | 97.55 | 97.53 | 97.55 | 0.00023 |
| **4** | 97.61 | 97.55 | 97.59 | 97.56 | 97.58 | 97.56 | 97.58 | 0.00023 |
| **6** | 97.54 | 97.50 | 97.54 | 97.55 | 97.54 | 97.55 | 97.54 | 0.00019 |
| **12** | 97.55 | 97.50 | 97.53 | 97.49 | 97.55 | 97.58 | 97.53 | 0.00034 |

**Table 11**

**The results of uncertainty evaluation of long-term stability**

| **Linear equation** | ***S*** | ***u_lts_*** |
| --- | --- | --- |
| y = -8.89×10^-6^x + 9.75×10^-1^ | 6.18×10^-4^ | 7.54×10^-4^ |

***4. Purity determination by mass balance method***

**Table 12**

**The results of purities determined by HPLC method**

| **No.** | **Purity (uncorrected**, **%)** | **Purity (corrected**, **%)** |
| --- | --- | --- |
| 1 | 97.48 | 95.76 |
| 2 | 97.28 | 95.43 |
| 3 | 97.15 | 95.20 |
| 4 | 97.49 | 95.80 |
| 5 | 97.50 | 95.79 |
| 6 | 97.65 | 96.02 |
| 7 | 97.29 | 95.43 |
| 8 | 97.68 | 96.09 |
| 9 | 97.30 | 95.40 |
| 10 | 97.50 | 95.78 |
| mean | 97.43 | 95.67 |
| Impurities |  | 1.05 |
| Purity |  | 94.67 |
| s |  | 0.000029 |

***5. Purity determination by quantitative nuclear magnetic resonance method***

**Table 13**

**The results of purities determined by qNMR**

| **No.** | Purity (%) |
| --- | --- |
| 1 | 95.92 |
| 2 | 95.88 |
| 3 | 95.98 |
| 4 | 95.61 |
| 5 | 95.78 |
| 6 | 95.48 |
| 7 | 95.65 |
| 8 | 95.82 |
| 9 | 96.22 |
| 10 | 95.59 |
| mean | 95.79 |
| Impurities | 1.05 |
| Purity | 94.74 |
| s | 0.0022 |

***6. u_CRM_ and U_CRM_ estimation***

**Table 14**

**The results of uncertainty evaluation of MB method**

|  | ***u*(*x*_oi_)** | ***u*(*x*_m_)** | ***u*(*x*_sa_)** | ***u*(*x*_r_)** |
| --- | --- | --- | --- | --- |
| ***Sal A*** | 1.50×10^-3^ | 2.47×10^-4^ | 2.04×10^-4^ | 2.02×10^-4^ |

**Table 15**

**The results of uncertainty evaluation of qNMR method**

|  | ***u*(*x*_qNMR_)** | ***u*(*x*_m_)** | ***u*(*x*_sa_)** | ***u*(*x*_r_)** |
| --- | --- | --- | --- | --- |
| ***Sal A*** | 6.99×10^-4^ | 2.47×10^-4^ | 2.04×10^-4^ | 2.02×10^-4^ |

**Table 16**

***The results of u_CRM_ and U_CRM_ estimation***

|  | ***u*_MB_** | ***u*_qNMR_** | ***u_h_*** | ***u_sts_*** | ***u_lts_*** | ***u_CRM_*** | ***U_CRM_*** |
| --- | --- | --- | --- | --- | --- | --- | --- |
| ***Sal A*** | 1.55×10^-3^ | 7.95×10^-4^ | 1.06×10^-4^ | 1.38×10^-3^ | 7.54×10^-4^ | 2.35×10^-3^ | 4.69×10^-3^ |

***7. Results of CRM analysis***

**Table 17**

***The results of CRM analysis***

|  | **Purity (%)** | ***U_CRM_* (%)** | ***k*** | ***P*** |
| --- | --- | --- | --- | --- |
| ***Sal A*** | 94.7 | 0.5 | 2 | 0.95 |

***6. Results of ICP-MS***

Microwave-assisted digestion

Approximately 250 mg of Sal A were directly weighted in PFA digestion vessels. A mixture comprising 1.0 mL of HNO_3_ (9.0 mol L-1), 3.0 mL of H_2_O_2_ (30%) and 6.0 mL of H_2_O was added to each vessel. Analytical blanks were prepared in the same way. The heating program was performed in four successive steps: (1) a two-minute ramp to reach 120 °C, (2) eight minutes hold at 120 °C, (3) a five-minute ramp to reach 180 °C, (4) fifteen minutes hold at 180 °C. In all steps, the oven was kept in 1600 W (maximum power). After the heating program, the vessels were cooled down for 15 min. Digested samples were diluted to 25.0 mL with ultrapure water (final digests). As a reference procedure to evaluate the digestion efficiency, an experiment was performed in triplicate, adding to the vessels 5.0 mL of HNO_3_ 65% and 2.0mL of H_2_O_2_ 30% using the same heating program established for diluted digestion.

**Table 18**

The results of CRM analysis

| **NO.** | **Sample Id** | **SAA** | **NO.** | **Sample Id** | **SAA** |
| --- | --- | --- | --- | --- | --- |
| **1** | B 11 (ppb) | 0.010 | **13** | Cu 63 (ppm) | ND |
| **2** | Na 23 (ppm) | 0.170 | **14** | Zn 66 (ppm) | 0.004 |
| **3** | Mg 24 (ppm) | ND | **15** | As 75 (ppb) | 0.050 |
| **4** | K 39 (ppm) | 0.020 | **16** | Se 82 (ppb) | ND |
| **5** | Ca 43 (ppm) | 0.235 | **17** | Rb 85 (ppb) | 0.060 |
| **6** | Ti 47 (ppb) | 0.100 | **18** | Sr 88 (ppb) | 0.602 |
| **7** | V 51 (ppb) | 0.234 | **19** | Mo 98 (ppb) | 0.052 |
| **8** | Cr 52 (ppb) | 12.323 | **20** | Cd 111 (ppb) | 0.002 |
| **9** | Mn 55 (ppb) | 0.289 | **21** | Sn 118 (ppb) | 1.046 |
| **10** | Fe 57 (ppm) | 0.006 | **22** | Ba 138 (ppb) | 1.224 |
| **11** | Co 59 (ppb) | 0.005 | **23** | Pb 208 (ppb) | ND |
| **12** | Ni 60 (ppb) | 0.350 |  |  |  |
